# Research Progress on Catalpol as Treatment for Atherosclerosis

**DOI:** 10.3389/fphar.2021.716125

**Published:** 2021-07-13

**Authors:** Lei He, Rusheng Zhao, Youheng Wang, Huibing Liu, Xuehui Wang

**Affiliations:** ^1^ Department of Cardiology, The First Affiliated Hospital of Xinxiang Medical University, Heart Center of Xinxiang Medical University, Xinxiang, China; ^2^ College of Life Science, Henan Normal University, Xinxiang, China

**Keywords:** atherosclerosis, catalpol, inflammation, oxidative stress, cell senescence, SIRT1

## Abstract

Coronary atherosclerotic heart disease, cerebrovascular disease, and peripheral artery disease are common diseases with high morbidity and mortality rates and must be addressed. Their most frequent complications, including myocardial infarction and stroke, are caused by spontaneous thrombotic occlusion and are the most frequent cause of death worldwide. Atherosclerosis (AS) is the most widespread underlying pathological change for the above diseases. Therefore, drugs that interfere with this pathophysiological process must be incorporated in the treatment. Chinese traditional and herbal drugs can effectively treat AS. With the development of traditional Chinese medicine, the active ingredients in common Chinese medicinal materials must be thoroughly purified prior to their application in western medicine. Various proprietary Chinese medicine preparations with remarkable effects have been used in AS treatment. Catalpol, the active component of *Rehmannia glutinosa*, belongs to iridoid terpene and has anti-inflammatory, antioxidant, insulin resistance improvement, and other related effects. Several reviews have been conducted on this compound and its actions against osteoporosis, neurodegenerative diseases, Alzheimer's disease (AD), Parkinson's disease (PD) and diabetes and its complications. The current review focused on catalpol’s effect on atherosclerotic plaque formation in different animal models. The potential mechanisms of catalpol to ameliorate AS were also summarized in terms of oxidative stress, inflammation, cell aging, apoptosis, and activation of the silent information regulator factor 2-related enzyme 1 (SIRT1) pathway.

## Introduction

Coronary atherosclerotic heart disease, cerebrovascular disease, and peripheral artery disease are common diseases with high morbidity and mortality rates and must be addressed. Their most common complications, including myocardial infarction and stroke, are caused by spontaneous thrombotic occlusion and are the most frequent cause of death worldwide (Kruk et al., 2018). AS is the most widespread underlying pathological change for these diseases (Gallino et al., 2014). Endothelial dysfunction is the primary prerequisite and important link for atherosclerotic plaque formation and is one of the important indicators for predicting the incidence and prognosis of cardio-cerebrovascular diseases (Daiber et al., 2017). For AS treatment, the reduction of low density lipoprotein cholesterol (LDL-C) level by statins and proprotein converting enzyme subtilisin/kexin 9 monoclonal antibody can reduce major cardiovascular adverse events by approximately 50% (Ridker et al., 2008). Angiotensin converting enzyme inhibitor (ACEI), angiotensin II type 1 receptor (AT1 receptor) antagonist, endothelin-1 receptor antagonist, and many other cardiovascular drugs show multiple indirect antioxidant properties and anti-inflammatory effects that can stabilize vascular endothelial function and ameliorate AS (Gori and Munzel, 2011; Steven et al., 2015). With the development of traditional Chinese medicine, the purification of active components in many common bulk medicinal materials in China has been further studied. Among which, catalpol, the active ingredient in *Rehmannia glutinosa* and a common medicinal material in China, has been widely explored for its pharmacological action.

**FIGURE 1:**
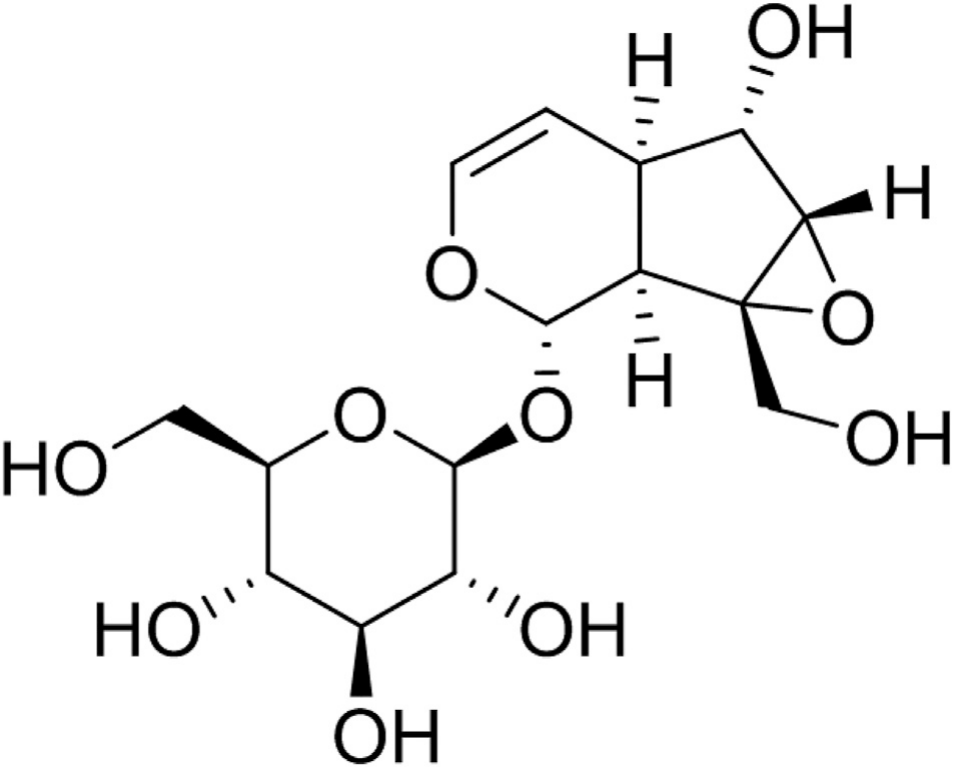
The chemical structure of catalpol.

Catalpol (C_15_H_22_O_10_, Figure 1 **.**) is an iridoid compound widely distributed in many plant families and is mainly extracted from the roots of *R. glutinosa,* one of the common bulk medicinal materials in China with the functions of nourishing yin, tonifying blood, enhancing essence, and tonifying marrow. Clinical trials for this plant have been conducted to verify its ameliorating effects on osteoporosis, such as increasing bone mineral density and maintaining the balance between osteoclasts and osteogenesis (Liu et al., 2017). The bone-protecting effect of *R. glutinosa* may be related to its high content of iridoid glycosides such as catalpol (Lim and Kim, 2013). Catalpol also has important neuroprotective effects on AD and PD (Dinda et al., 2019), exhibits anti-diabetic activity on different animal models, and prevents diabetic nephropathy, heart, central nervous system, and bone complications (Bai et al., 2019). Several reviews have been conducted on its actions against osteoporosis, neurodegeneration, AD, PD, and diabetes and its complications. This article summarized the effect and mechanism of catalpol against AS.

## Animal and Cell Models Used in Research on Catalpol for Atherosclerosis Treatment

Various atherosclerotic models have been used to prove the effect of catalpol on atherosclerotic plaque formation. Table 1lists the reviewed *in vivo* AS models, drug administration routes, catalpol dosages, and intervention times. The models include the following: 1) the AS model induced in male New Zealand white rabbits by feeding high cholesterol diet (containing 1% cholesterol), 2) the diabetic AS model in diabetic rabbits induced by high fat diet combined with intravenous injection of alloxan, 3) the AS model in LDLr^−/−^ mice induced by high fat diet, and 4) the AS model in ApoE^−/−^ mice induced by high fat diet combined with ovariectomy. Oral administration was generally used in the studies. Catalpol dosage varied for different models, that is, 5 mg/kg/day for 4 month-old male New Zealand white rabbits, 100–200 mg/kg/day for LDLr^−/−^ male mice, and 20 mg/kg/day for ApoE^−/−^ female mice. Endothelial dysfunction is a preliminary pathophysiological event in AS development (Suwaidi et al., 2000). Macrophages and smooth muscle cells migrate, proliferate, differentiate, and phagocytize lipids into foam cells, an essential link of AS plaque formation. Table 2 shows the construction of *in vitro* model and the concentration and treatment time of catalpol.

**TABLE 1:** Different animal models have been used to prove that catalpol improves AS.

As *in vivo* model	Drug administration route	Catalpol dose	Processing time	Reference
High cholesterol rabbit	Oral administration	5 mg/kg/d	12 w	Liu and Zhang. (2015)
Diabetic rabbit	Oral administration	5 mg/kg/d	12 w	Liu et al. (2016)
LDLr^−/−^ mice + HFD^1^	Oral administration	100 mg/kg/d	16 w	Zhang et al. (2018)
		200 mg/kg/d		
ApoE^−/−^mice + HFD^2^+ Ovx	Oral administration	20 mg/kg/d	90 d	Chen et al. (2021)

Note: High cholesterol rabbit (induced by feeding rabbit feed containing 1% cholesterol); Diabetic rabbit (induced by intravenous injection of alloxan 100 mg/kg after 4 weeks of high-fat diet containing 1% cholesterol and 10% lard); LDLr^−/−^ mice:low density lipoprotein receptor knockout mice; HFD^1^ (high-fat diet: contains 20.0% cocoa fat, 1.25% cholesterol, 22.5% protein and 45.0% carbohydrates); ApoE^**−/−**^ mice: apolipoprotein E-knockout mice; HFD^2^ (high-fat diet: containing 0.3% cholesterol and 20% pork fat); Ovx: ovariectomy.

**TABLE 2:** Different cell models were used to explore the potential mechanism of catalpol in improving AS.

As *in vitro* model	Damage factors	Catalpol dose	Processing time (h)	Reference
EA.hy926	oxLDL (100 μg/ml)	50 μM	2	Liu and Zhang. (2015)
THP-1	oxLDL (100 μg/ml)	5–80 μM	24	Zhang et al. (2018)
HAECs	HCY (3 mM)	7.5, 15,30 μM	24	Hu et al. (2019)
THP-1	LPS (1 μg/ml)	50 μM	24	Zhu et al. (2014)
MH-S	LPS (1 μg/ml)	0.1–0.5 mM	48	Fu et al. (2014)
HaCaT	TNF-α (10 ng/ml)	7.5, 15,30 μM	24	Liu et al. (2021)
MPC-5	ADR (1 μM)	2,4,8 μM	48	Zhang et al. (2019)
HUVECs	H_2_O_2_ (100 μM)	0.1–10 μg/ml	24	Hu et al. (2010)
IEC-6	Brefeldin A (1 μM)	5–80 μM	24	Xiong et al. (2017)
J774A-1	LPS (100 ng/ml)	5, 10, 20 μM	24	Chen et al. (2021)
	IFN-γ(20 ng/ml)			

Note: EA.hy926 belongs to human endothelial cell line; THP-1: human monocytic leukemia cell line; Ox-LDL: oxidized low density lipoprotein; HAECs: human aortic endothelial cells; HCY: homocysteine; LPS: lipopolysaccharide; MH-S: mouse alveolar macrophage line; HaCaT: keratinocyte line; ADR: adriamycin; MPC-5: mouse podocyte line; HUVECs: human umbilical vein endothelial cells; H_2_O_2_: hydrogen peroxide; IEC-6: rat intestinal epithelial cell; Brefeldin A: specific endoplasmic reticulum stress inducer; J774A-1 belongs to the mouse macrophage cell line; IFN-γ: interferon-γ.

Using the atherosclerotic model of rabbits with high cholesterol, Liu et al. observed the area of aortic AS and found a lesion in the high cholesterol chow (HCC) group accounting for 54% of the total area but not in the normal diet group. Compared with the HCC group, the catalpol and atorvastatin groups had reductions in the area of atherosclerotic lesions by 58 and 45%, respectively. In addition, oral catalpol administration for 12 weeks (5 mg/kg/day) substantially inhibited neointimal hyperplasia and the infiltration of smooth muscle cells and macrophages in plaques (Liu and Zhang., 2015). Zhang et al. fed LDLr^−/−^ mice with high-fat diet for 16 weeks and stripped the aorta for oil red staining, HE staining, and immunohistochemical staining. In the HFD group, the area of subintimal lesion was enlarged and contained a necrotic core rich in foam cells derived from macrophages. In the catalpol intervention group, the number of foam cells derived from subendothelial macrophages was decreased. This effect became highly pronounced when the dose of catalpol was 200 mg/kg/day (Zhang et al., 2018). Another study showed that catalpol can reduce the AS of alloxan-induced diabetic rabbits and delay AS progression (Liu et al., 2016). In a recent study simulating postmenopausal AS, female ApoE^−/−^ mice were ovariectomized and fed with a high-fat diet to induce atherosclerotic plaque formation and stop estrogen production to mimic postmenopausal behavior. The area of atherosclerotic plaque in the oral catalpol group was smaller than that in the ovariectomized high-fat diet group. Catalpol also promoted the expression of estrogen receptors (ERs) in HFD-ovariectomy (Ovx)-treated ApoE^−/−^ mice (Chen et al., 2021).

## Potential Mechanism of Catalpol in Improving Atherosclerosis

Catalpol is a naturally occurring 7, 8-cyclopentane iridoid and one of the metabolites formed by the fusion of cyclopentane ring and pyran ring skeleton (including acetal structure) mainly in the form of glycosides. Catalpol improves AS plaque formation. Discussion on its potential mechanism is helpful to further understand the biological role of catalpol and find evidence to support its clinical application.

### Catalpol Plays an Anti-AS Role by Inhibiting Oxidative Stress and Endoplasmic Reticulum Stress

OS is one of the most common pathophysiological processes in AS formation (Libby et al., 2011). Catalpol decreases the concentration of oxidative factors including malondialdehyde, protein carbonyl, advanced glycation end products, and oxidized LDL-C in the serum and liver of hypercholesterolemic rabbit AS, diabetic rabbit AS, and hyperlipidemic AS models. However, this compound also increases the concentration of some antioxidant factors such as superoxide dismutase and glutathione peroxidase and the total antioxidant capacity (Liu and Zhang, 2015; Liu et al., 2016; Zhang et al., 2018). Relevant OS indicators were detected to reflect the level of oxidation *in vivo*, and the results verified that catalpol could partially improve AS plaque formation by reducing OS degree.

Nuclear factor erythroid related factor 2 (Nrf2) is an important redox sensitive transcription factor in cellular antioxidant defense. Under OS, Nrf2 enters the nucleus and upgrades various antioxidants, including heme oxygenase (HO-1). Liu et al. found that catalpol promotes Nrf2 nuclear translocation and HO-1 expression in EA.hy926 cells, thus implying its antioxidant role (Liu and Zhang, 2015).

Nicotinamide adenine dinucleotide phosphate (NADPH) oxidase family composed of nitrogenoxide (NOX) 1-5 is the most important source of ROS. P22^phox^ is an important component of the NADH/NADPH oxidase system that produces superoxides (Ushio-Fukai et al., 1996). Zhang et al. reported that catalpol has a potential inhibitory effect on P22, NOX2, and NOX4 protein expression and ROS production in AS mice and oxLDL-treated macrophages (Zhang et al., 2018).

ER is an important organelle that regulates several basic cellular processes, controls all important connections to the cellular external environment, and responds to systemic metabolism, inflammation, and endocrine and mechanical stimuli (Ochoa et al., 2018). Chronic ER stress is induced by various *in vivo* factors, interact with OS and inflammasome activation, and finally aggravate AS formation (Ochoa et al., 2018). The molecules commonly used to reflect the OS level in the body include protein kinase R-like endoplasmic reticulum kinase (PERK), activated transcription factor 6, IRE1 α, and GRP78.

The increase in the pathophysiological level of homocysteine (HCY) in circulation is related to the accelerated production of superoxide and peroxynitrite and the inhibited antioxidant defense of cardiovascular system (Suematsu et al., 2007). HCY can trigger ER stress by interfering with the formation of disulfide bonds and inducing unfolded protein response (Yu et al., 2013). Severe and persistent ER stress mediates the apoptosis induced by CHOP, JNK, and caspase-12 (Lu et al., 2014; Sisinni et al., 2019). Hu et al. found that catalpol reduces the level of OS and ER stress induced by HCY in human aortic endothelial cells, thus partly protecting endothelial cells from HCY toxicity by inhibiting NOX4 expression and blocking NF-κB/p65 signal pathway (Hu et al., 2019).

### Catalpol Reduces the Degree of Inflammatory Response in Atherosclerosis

Inflammation runs through AS formation and plays an important role in every link of this condition. NF-κB activation is related to the expression of many pro-inflammatory cytokines, inflammatory cytokines, and adhesion molecules, such as interleukin-6 (IL-6), tumor necrosis factor-α (TNF-α), monocyte chemoattractant protein-1 (MCP-1), intercellular adhesion molecule-1 (ICAM-1), vascular cell adhesion molecule-1 (VCAM-1), inducible nitric oxide synthase (iNOS), and matrix metalloproteinase-9 (MMP-9) (Randolph, 2014). These cytokines promote the adhesion of neutrophils and monocytes to the endothelium and the remodeling of extracellular matrix, thus affecting the production of endothelium-derived nitric oxide (NO), reducing endothelium-dependent vasodilation, and finally initiating AS aggravation.

Liu et al. found that catalpol alleviates the inflammatory response of AS in hypercholesterolemic rabbits by reducing the levels of circulating TNF-α, IL-6, soluble VCAM-1, soluble ICAM-1, and MCP-1 and the expression levels of VCAM-1, MCP-1, TNF- α, iNOS, MMP-9, and nuclear factor-κ B protein 65 (NF- κ B p65) in thoracic (Liu and Zhang, 2015). Catalpol also inhibits I-κB protein degradation, NF-κB trans-activation, and NF-κB p65 binding activation in oxLDL-induced EA.hy926 cells, suggesting its regulating role in inflammation through the NF-κB-dependent pathway (Liu and Zhang, 2015). In their other study, catalpol suppressed alloxan-induced inflammation in diabetic rabbits by reducing the overexpression of TNF- α, MCP-1, and VCAM-1 during circulation (Liu et al., 2016).

Chen et al. found that catalpol inhibits the increased polarization, inflammatory response, and oxidative stress of macrophages induced by lipopolysaccharides (LPS) and interferon-γ (IFN-γ) and improves the function of macrophages activated by classical pathways. A postmenopausal mouse AS model was established by ovariectomizing the treated ApoE^−/−^ mice and feeding them with a high-fat diet. The serum of ApoE^−/−^ mice was collected in the *in vivo* experiment. The levels of proinflammatory cytokines (C-reactive protein, TNF-α, and IL-1β) were decreased, whereas those of anti-inflammatory cytokines (IL-10) were increased in the catalpol-treated group. These protective effects were inhibited after Erα was blocked, indicating that catalpol exerts its anti-inflammatory effects by regulating ERα (Chen et al., 2021).

Nucleotide binding domain-like receptor protein 3 (NLRP3) inflammatory body is a multiprotein complex that combines with ASC (apoptosis-related spot-like protein containing caspase1 activation domain, CARD) and caspase-1 to regulate the maturation of pro-inflammatory cytokines IL-1 β and IL-18 (Kelley et al., 2019). Zhu et al. found that catalpol treatment could reduce the expression of LPS-induced inflammatory body NLRP3 and then inhibit the mRNA and protein levels of pro-inflammatory cytokines such as IL-1 β, pro-IL-1 β, TNF-α, and IL-32, thus improving the inflammatory response in THP-1 cells (Zhu et al., 2014). By using the *in vitro* model of acute lung injury induced by LPS in MH-S alveolar macrophages, Fu et al. found the decreased expression of inflammatory cytokines TNF- α, IL-6, IL-4, and IL-1 β induced by LPS after treatment with different catalpol (0.1–0.5 mM) concentrations, thereby proving that catalpol has an anti-inflammatory effect (Fu et al., 2014).

SIRT1 deletion in endothelial cell (ECs), vascular smooth muscle cells (VSMCs), and monocytes/macrophages can increase OS, inflammation, foam cell formation, aging damage nitric oxide production, and autophagy and thus promote vascular aging and AS (Kitada et al., 2016). Liu et al. found that catalpol inhibits NF-κB and MAPK signaling pathways by up-regulating the expression of SIRT1 mRNA and consequently reducing OS and TNF-α-induced inflammation response (Liu et al., 2021). Endogenous SIRT1 remarkably reduces endothelial activation without affecting the vasoconstriction and dilation function of ApoE−/− mice. Continuous endothelial activation by various risk factors leads to the occurrence and development of AS. Therefore, SIRT1 activation can produce an anti-atherosclerotic effect (Stein et al., 2010). Zhang et al. used molecular docking simulation to explore the bioactive conformations of catalpol and common SIRT1 activators (resveratrol, SRT2140, and quercetin) with SIRT1. Their total integral values were 6.4519, 4.1586, 6.0038, and 5.4237, respectively. These results showed that the bioactive conformations of catalpol and SIRT1 have higher total integral values than other SIRT1 activators, indicating that catalpol has higher affinity with SIRT1 than other common SIRT1 activators and is likely to become a SIRT1 agonist (Zhang et al., 2019). Exploration of the mutual regulation relationship between catalpol and SIRT1 proved that microRNA-132 (miR-132) regulates the inflammatory process by negatively controlling SIRT1 expression (Varma-Doyle et al., 2021). Xiong et al. reported that catalpol can resist ER stress induced by breviscapine A, a specific ER stress inducer, by activating SIRT1 through miR-132 down-regulation. The protective effect of catalpol is concentration-dependent (Xiong et al., 2017). Based on the above findings, our experimental group is currently conducting a study entitled “catalpol exerts its anti-AS effect by activating SIRT1” to further explore the clinical application feasibility and related protective mechanism of catalpol in AS.

### Catalpol Slows Down the Progress of Vascular Cell Senescence in Atherosclerosis

Atherosclerotic plaques show characteristics of cell aging, such as reduced cell proliferation (Bennett et al., 1995), irreversible growth arrest and apoptosis (Lutgens et al., 1999), increased DNA damage (Botto et al., 2001), epigenetic modification (Lund and Zaina, 2011), and shortening and dysfunction of telomeres (Matthews et al., 2006; Ogami et al., 2004). Telomere length and integrity are regulated by telomerase interaction (De Lange, 2005). Telomerase reverse transcriptase (TERT) maintains the telomere terminal by catalyzing the increase in short telomere repeat sequence during DNA replication (Bressler et al., 2015). Zhang et al. used the comet test and measured 8-hydroxydeoxyguanosine (8-OHdG) levels to indirectly reflect the extent of DNA damage. *In vivo* and *in vitro* results verified that catalpol can reduce the level of 8-OHdG and shorten the length of comet tail, indicating its partial mitigating action on the degree of DNA oxidative damage. In addition, the telomerase activity of macrophages decreased after treatment with oxLDL. Catalpol could concentration-dependently increase the telomerase activity and partially inhibit the senescence of macrophages (Zhang et al., 2018). Peroxisome proliferator-activated receptor-γ coactivator α (PGC-1 α) is a key molecule connecting OS and telomere function (Miao et al., 2016). PGC-1 α upregulation in aortic smooth muscle cells can enhance telomere function, reduce DNA damage, and thus inhibit AS (Xiong et al., 2015). Zhang et al. reported that in the AS model of LDLr^−/−^ mice induced by high-fat diet, the expression of PGC-1α and TERT decreased to 37 and 45%, respectively, in the HFD group. On the contrary, the expression levels of PGC-1 α and TERT protein were substantially up-regulated in the catalpol group. oxLDL-treated macrophages were used to form a cell injury model by applying the cell transfection technique. The results proved that catalpol reduces ROS accumulation and DNA damage and improves telomere function through the PGC-1 α/TERT pathway. Zhang et al. reported that catalpol can directly enhance the activity of PGC-1 α promoter as indicated by the luciferase activity assay (Zhang et al., 2018).

### Catalpol Plays an Anti-apoptotic Role in Atherosclerosis

Bcl-2 family members located on the mitochondrial membrane can change mitochondrial permeability and eventually induce apoptosis (Zamzami et al., 1998). Some members of the Bcl-2 family induce cell survival (Bcl-2 and Bcl-xL) or promote apoptosis (Bad and Bax) (Datta et al., 1997; Olivetti et al., 1997). Using the AS model of LDLr^−/−^ mice fed with high-fat diet, Zhang et al. found that catalpol upregulates the protein level of anti-apoptotic protein B-cell lymphoma-2 (Bcl-2) and downregulates the level of apoptotic protein caspase3 and caspase9, thereby suggesting its potential anti-apoptotic effect (Zhang et al., 2018). Intracellular signaling pathway-Akt is involved in the regulation of cell survival and apoptosis. Its activation phosphorylates Bad and thus inhibit cell apoptosis (Datta et al., 1997). Hu et al. reported that after HUVECs were treated with 100 μM H_2_O_2_ for 24 h, apoptotic protein Bax was up-regulated, Bad phosphorylation was inactivated, and anti-apoptotic protein Bcl-2 was down-regulated, thus resulting in an increased rate of apoptosis. However, catalpol could also play an anti-apoptotic effect by activating Akt to phosphorylate Bad, down-regulate Bax, and up-regulate Bcl-2. These protective effects can be partially inhibited by phosphatidylinositol 3-kinase (PI3K) antagonist (Wortmannin or LY294002). Therefore, catalpol may partially regulate the Bcl-2 family through the PI3K/Akt pathway and then protect HUVECs from H_2_O_2_ toxicity (Hu et al., 2010).

## Efficacy and Safety of Catalpol

Acute toxicity experiments related to catalpol have been conducted in mouse models to determine its safety (Bai et al., 2019). The 50% lethal dose of catalpol in mice was determined as 206.5 mg/kg after intraperitoneal catalpol injection. After a long-term intravenous catalpol injection (10, 20, and 40 mg/kg/day; 90 days), no toxic changes were observed in the biochemical indexes and physiological structure of rat organs (Jang et al., 2008). This finding indicated that catalpol has no side effects. A preliminary clinical experimental study explored the safety of catalpol treatment in patients with colon cancer by intraperitoneally injecting 10 mg/kg catalpol twice a day for 12 weeks. Only mild non-fatal adverse reactions, such as nausea, vomiting, gastrointestinal ulcers, and constipation, were observed (Fei et al., 2018). Therefore, catalpol performs well in terms of safety. With regard to its effectiveness, four independent *in vivo* experiments revealed that this compound could substantially reduce the area of subintimal atherosclerotic plaque and inhibit the thickening of neointima and the formation of foam cells. In many *in vitro* models, catalpol reduces cell injury and improves cell function through its anti-inflammatory, anti-oxidative stress, anti-apoptosis, and other effects. Therefore, this ingredient shows efficacy for AS treatment to a certain extent. The specific protective mechanism, efficacy, and safety of catalpol for AS treatment still need to be further explored and improved. As an effective ingredient of *R. glutinosa*, catalpol is expected to be a new drug for AS treatment.

## Pharmacokinetics of Catalpol

Differences in pharmacokinetics were observed for catalpol administered through various routes. The bioavailability of intramuscular injection was 71.63%, whereas that of oral administration was only 49.38% (Bai et al., 2019). This difference may be related to its chemical properties, that is, catalpol is easily destroyed in acidic environment and is relatively stable in alkaline condition. This phenomenon confirmed the instability of catalpol in the gastrointestinal tract. In all the animal models in this review, catalpol was administered through oral administration. The results also showed that catalpol had a good anti-AS effect. The reason may be related to the production of some active metabolites of catalpol after metabolic transformation *in vivo*. UHPLC-Q-Exactive MS was used to analyze the metabolites of catalpol in rats, and 29 metabolites (including catalpol) were detected, including 19 metabolites in urine, 3 metabolites in plasma, and 14 metabolites in feces (Xiang et al., 2021). Most of these metabolites existed in the form of catalpol aglycone. Investigation on its biotransformation pathway revealed that catalpol was first transformed into catalpol aglycone through deglycosylation after oral administration. A series of phase I metabolic reactions subsequently occurred, including hydroxylation, dihydroxylation, hydrogenation, dehydrogenation, and oxidation of methylene to ketone, followed by phase II metabolic reactions, including glucuronidation and glycine and cysteine conjugation.

Abundant metabolites were detected in rat feces. This finding indicated the presence of copious intestinal microflora in the intestinal tract, which might be one of the main metabolic organs of catalpol. Another study speculated that catalpol id first metabolized into catalpol aglycone by β-D-glucosidase from different human intestinal bacteria; catalpol aglycone is then metabolized into acetylated, demethylated, and hydroxylated catalpol (Tao et al., 2016).

Catalpol may be the precursor of the above active metabolites. Biotransformation has its unique advantages over traditional prodrug transformation. Although catalpol is unstable in the gastrointestinal tract, oral administration of this drug is still the most common route. Unique biotransformation to metabolize catalpol into active metabolites could be applied to ensure its exceptional biological role.

## Conclusion

AS is the most common pathophysiological process in coronary atherosclerotic heart disease, cerebrovascular disease, and peripheral artery disease, all of which have high morbidity and mortality. Given that statins regulate lipid metabolism, ACEI, AT1 receptor antagonists, and other drugs. Therefore, the application of Chinese medicine adjunctive therapy for AS is also an essential link. At present, many kinds of Chinese patent medicines, including “Xuezhikang,” “Shensong Yangxin Capsule,” “Compound Danshen Dropping Pill,” “Tongxinluo,” and “Xuesaitong”, have been used in the clinical adjuvant treatment of coronary atherosclerotic heart disease. With the wide clinical application of traditional Chinese medicines and their good curative effect, their Western usage is currently on a gradual rise. Further exploration on their effective ingredients of traditional Chinese medicine for clinical application has become the top priority to carry forward the culture of traditional Chinese medicine.

**FIGURE 2:**
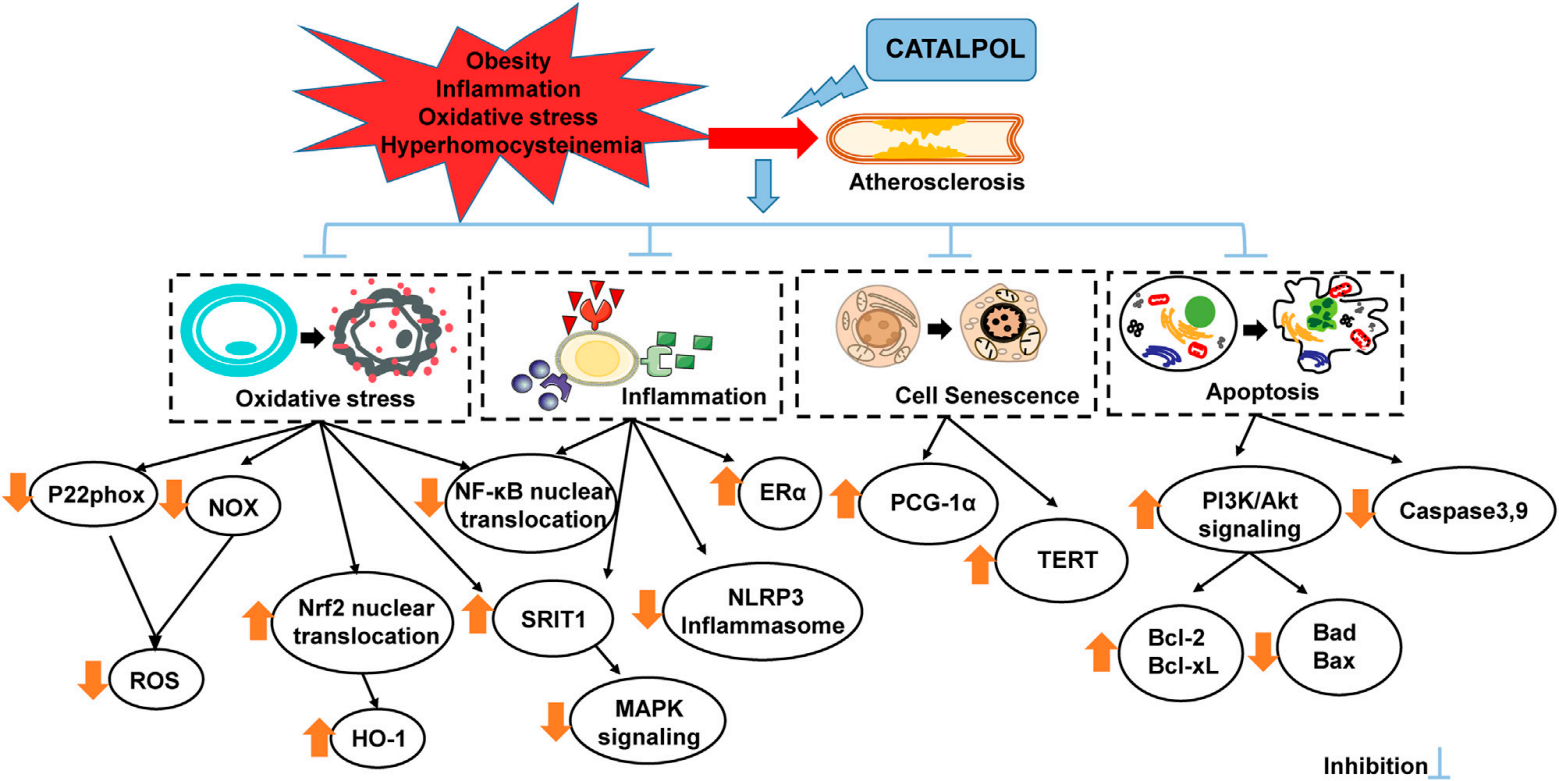
The detailed mechanism of catalpol’s protective effect on AS.

Catalpol, the active ingredient of *R. glutinosa*, has been widely studied. Through its anti-inflammation, antioxidation, ER stress, cell injury reduction, and anti-apoptosis effects *in vivo* and *in vitro*, this compound can improve the degree of vascular cell injury, atherosclerotic plaque area, media thickness, plaque lipid content, and vascular smooth muscle cell and macrophage infiltration in different AS models (Figure 2 **.**) However, the clinical application of catalpol for coronary atherosclerotic heart disease still require further exploration, particularly its safety and efficacy.

## Glossary

AS: Atherosclerosis
AGEs: advanced glycation end products
ATF6: Activating Transcription Factor6
ER: endoplasmic reticulum
ERs: estrogen receptors
GSH: glutathione
GSH-Px: glutathione peroxidase
HCY: Homocysteine
HHCY: hyper-homocysteinemia
HDL-C: high density lipoprotein cholesterol
HO-1: heme oxygenase
ICAM-1: intercellular adhesion molecule-1
IL: interleukin
IRE1 α: Inositol-requiring transmembrane kinase/endonuclease-1
iNOS: inducible nitric oxide synthase
IFN-γ: interferon-γ
JNK: c-Jun NH2-terminal kinase
LDL-C: low density lipoprotein cholesterin
LDH: lactate dehydrogenase
LPS: lipopolysaccharides
MMP: matrix metalloproteinase
MDA: malondialdehyde
MCP-1: monocyte chemoattractant protein-1
MMP-9: matrix metalloproteinases-9
MAPK: mitogen-activated protein kinase
Nrf2: nuclear factor erythroid 2-related factor 2
NOX: nitrogen oxide
NADPH: nicotinamide adenine dinucleotide phosphate
NF-κB p65: nuclear factor-κB protein65
NF-κB: nuclear factor-κB
NLRP3: nucleotide-binding domain-like receptor protein 3
ox-LDL: oxidized low density lipoprotein cholesterin
OS: oxidative stress
PDGF: platelet-derived growth factor
PCG: protein carbonyl groups
PGC-1α: Peroxisome proliferator-activated receptor-γ coactivator α
PERK: proteinkinaseR—likeERkinase
PI3K: phosphatidylinositol 3-kinase
ROS: Reactive oxygen species
SOD: superoxide dismutase
sVCAM-1: soluble vascular cell adhesion molecule-1
sICAM-1: soluble intercellular adhesion molecule-1
TNF-α: tumor necrosis factor-α
TAS: total antioxidant status
TERT: telomerase reverse transcriptase
UPR: unfolded protein response
VCAM-1: vascular cell adhesion molecule-1
8-OHdG: 8-Hydroxydeoxyguanosine

## References

[B1] Bai Y. Zhu R. Tian Y. Li R. Chen B. Zhang H. (2019). Catalpol in Diabetes and its Complications: A Review of Pharmacology, Pharmacokinetics, and Safety. Molecules 24, 3302. 10.3390/molecules24183302 PMC6767014 31514313

[B2] Bennett M. R. Evan G. I. Schwartz S. M. (1995). Apoptosis of Human Vascular Smooth Muscle Cells Derived from normal Vessels and Coronary Atherosclerotic Plaques. J. Clin. Invest. 95, 2266–2274. 10.1172/jci117917 7738191 PMC295839

[B3] Botto N. Rizza A. Colombo M. G. Mazzone A. M. Manfredi S. Masetti S. (2001). Evidence for DNA Damage in Patients with Coronary Artery Disease. Mutat. Research/Genetic Toxicol. Environ. Mutagenesis 493, 23–30. 10.1016/s1383-5718(01)00162-0 11516712

[B4] Bressler J. Franceschini N. Demerath E. W. Mosley T. H. Folsom A. R. Boerwinkle E. (2015). Sequence Variation in Telomerase Reverse Transcriptase (TERT) as a Determinant of Risk of Cardiovascular Disease: the Atherosclerosis Risk in Communities (ARIC) Study. BMC Med. Genet. 16, 52. 10.1186/s12881-015-0194-x 26201603 PMC4557920

[B5] Chen Q. Qi X. Zhang W. Zhang Y. Bi Y. Meng Q. (2021). Catalpol Inhibits Macrophage Polarization and Prevents Postmenopausal Atherosclerosis through Regulating Estrogen Receptor Alpha. Front. Pharmacol. 12, 655081. 10.3389/fphar.2021.655081 33995075 PMC8120111

[B6] Daiber A. Steven S. Weber A. Shuvaev V. V. Muzykantov V. R. Laher I. (2017). Targeting Vascular (Endothelial) Dysfunction. Br. J. Pharmacol. 174, 1591–1619. 10.1111/bph.13517 27187006 PMC5446575

[B7] Datta S. R. Dudek H. Tao X. Masters S. Fu H. Gotoh Y. (1997). Akt Phosphorylation of BAD Couples Survival Signals to the Cell-Intrinsic Death Machinery. Cell 91, 231–241. 10.1016/s0092-8674(00)80405-5 9346240

[B8] De Lange T. (2005). Shelterin: the Protein Complex that Shapes and Safeguards Human Telomeres. Genes Development 19, 2100–2110. 10.1101/gad.1346005 16166375

[B9] Dinda B. Dinda M. Kulsi G. Chakraborty A. Dinda S. (2019). Therapeutic Potentials of Plant Iridoids in Alzheimer's and Parkinson's Diseases: A Review. Eur. J. Med. Chem. 169, 185–199. 10.1016/j.ejmech.2019.03.009 30877973

[B10] Fei B. Dai W. Zhao S. (2018). Efficacy, Safety, and Cost of Therapy of the Traditional Chinese Medicine, Catalpol, in Patients Following Surgical Resection for Locally Advanced Colon Cancer. Med. Sci. Monit. 24, 3184–3192. 10.12659/msm.907569 29763415 PMC5975072

[B11] Fu K. Piao T. Wang M. Zhang J. Jiang J. Wang X. (2014). Protective Effect of Catalpol on Lipopolysaccharide-Induced Acute Lung Injury in Mice. Int. Immunopharmacology 23, 400–406. 10.1016/j.intimp.2014.07.011 25063711

[B12] Gallino A. Aboyans V. Diehm C. Cosentino F. Stricker H. Falk E. (2014). Non-coronary Atherosclerosis. Eur. Heart J. 35, 1112–1119. 10.1093/eurheartj/ehu071 24595865

[B13] Gori T. Münzel T. (2011). Oxidative Stress and Endothelial Dysfunction: Therapeutic Implications. Ann. Med. 43, 259–272. 10.3109/07853890.2010.543920 21284528

[B14] Hu H. Wang C. Jin Y. Meng Q. Liu Q. Liu Z. (2019). Catalpol Inhibits Homocysteine-Induced Oxidation and Inflammation via Inhibiting Nox4/NF-Κb and GRP78/PERK Pathways in Human Aorta Endothelial Cells. Inflammation 42, 64–80. 10.1007/s10753-018-0873-9 30315526 PMC6394570

[B15] Hu L. Sun Y. Hu J. (2010). Catalpol Inhibits Apoptosis in Hydrogen Peroxide-Induced Endothelium by Activating the PI3K/Akt Signaling Pathway and Modulating Expression of Bcl-2 and Bax. Eur. J. Pharmacol. 628, 155–163. 10.1016/j.ejphar.2009.11.046 19962976

[B16] Jang T. Zhang A. Zhao R. Jang B. (2008). Protective Effect of Catalpol in Mice Injuries Induced by Rotenone and Evaluation of the Safety of Catalpol. Prog. Mod. Biomed. 8, 1039–1041+1045. 10.13241/j.cnki.pmb.2008.06.016

[B17] Kelley N. Jeltema D. Duan Y. He Y. (2019). The NLRP3 Inflammasome: An Overview of Mechanisms of Activation and Regulation. Ijms 20, 3328. 10.3390/ijms20133328 PMC6651423 31284572

[B18] Kitada M. Ogura Y. Koya D. (2016). The Protective Role of Sirt1 in Vascular Tissue: its Relationship to Vascular Aging and Atherosclerosis. Aging 8, 2290–2307. 10.18632/aging.101068 27744418 PMC5115889

[B19] Kruk M. E. Gage A. D. Joseph N. T. Danaei G. García-Saisó S. Salomon J. A. (2018). Mortality Due to Low-Quality Health Systems in the Universal Health Coverage Era: a Systematic Analysis of Amenable Deaths in 137 Countries. The Lancet 392, 2203–2212. 10.1016/s0140-6736(18)31668-4 PMC6238021 30195398

[B20] Libby P. Ridker P. M. Hansson G. K. (2011). Progress and Challenges in Translating the Biology of Atherosclerosis. Nature 473, 317–325. 10.1038/nature10146 21593864

[B21] Lim D. Kim Y. (2013). Dried Root of Rehmannia Glutinosa Prevents Bone Loss in Ovariectomized Rats. Molecules 18, 5804–5813. 10.3390/molecules18055804 23685937 PMC6270096

[B22] Liu A. Zhang B. Zhao W. Tu Y. Wang Q. Li J. (2021). Catalpol Ameliorates Psoriasis-like Phenotypes via SIRT1 Mediated Suppression of NF-Κb and MAPKs Signaling Pathways. Bioengineered 12, 183–195. 10.1080/21655979.2020.1863015 33323018 PMC8806253

[B23] Liu C. Ma R. Wang L. Zhu R. Liu H. Guo Y. (2017). Rehmanniae Radix in Osteoporosis: A Review of Traditional Chinese Medicinal Uses, Phytochemistry, Pharmacokinetics and Pharmacology. J. Ethnopharmacology 198, 351–362. 10.1016/j.jep.2017.01.021 28111216

[B24] Liu J.-y. Zhang D.-j. (2015). Amelioration by Catalpol of Atherosclerotic Lesions in Hypercholesterolemic Rabbits. Planta Med. 81, 175–184. 10.1055/s-0034-1396240 25671384

[B25] Liu J. Y. Zheng C. Z. Hao X. P. Zhang D. J. Mao A. W. Yuan P. (2016). Catalpol Ameliorates Diabetic Atherosclerosis in Diabetic Rabbits. Am. J. Transl Res. 8, 4278–4288. 27830011 PMC5095320

[B26] Lu J.-p. Li X. Jin Y.-l. Chen M.-x. (2014). Endoplasmic Reticulum Stress-Mediated Aldosterone-Induced Apoptosis in Vascular Endothelial Cells. J. Huazhong Univ. Sci. Technol. [Med. Sci. 34, 821–824. 10.1007/s11596-014-1359-0 25480576

[B27] Lund G. Zaina S. (2011). Atherosclerosis: an Epigenetic Balancing Act that Goes Wrong. Curr. Atheroscler. Rep. 13, 208–214. 10.1007/s11883-011-0174-3 21384259

[B28] Lutgens E. De Muinck E. D. Kitslaar P. J. Tordoir J. H. Wellens H. J. Daemen M. J. (1999). Biphasic Pattern of Cell Turnover Characterizes the Progression from Fatty Streaks to Ruptured Human Atherosclerotic Plaques. Cardiovasc. Res. 41, 473–479. 10.1016/s0008-6363(98)00311-3 10341847

[B29] Matthews C. Gorenne I. Scott S. Figg N. Kirkpatrick P. Ritchie A. (2006). Vascular Smooth Muscle Cells Undergo Telomere-Based Senescence in Human Atherosclerosis. Circ. Res. 99, 156–164. 10.1161/01.RES.0000233315.38086.bc 16794190

[B30] Miao G.-Y. Zhou X. Zhang X. Xie Y. Sun C. Liu Y. (2016). Telomere-Mitochondrion Links Contribute to Induction of Senescence in MCF-7 Cells after Carbon-Ion Irradiation. Asian Pac. J. Cancer Prev. 17, 1993–1998. 10.7314/apjcp.2016.17.4.1993 27221886

[B31] Ochoa C. D. Wu R. F. Terada L. S. (2018). ROS Signaling and ER Stress in Cardiovascular Disease. Mol. Aspects Med. 63, 18–29. 10.1016/j.mam.2018.03.002 29559224 PMC6139279

[B32] Ogami M. Ikura Y. Ohsawa M. Matsuo T. Kayo S. Yoshimi N. (2004). Telomere Shortening in Human Coronary Artery Diseases. Atvb 24, 546–550. 10.1161/01.ATV.0000117200.46938.e7 14726417

[B33] Olivetti G. Abbi R. Quaini F. Kajstura J. Cheng W. Nitahara J. A. (1997). Apoptosis in the Failing Human Heart. N. Engl. J. Med. 336, 1131–1141. 10.1056/nejm199704173361603 9099657

[B34] Randolph G. J. (2014). Mechanisms that Regulate Macrophage burden in Atherosclerosis. Circ. Res. 114, 1757–1771. 10.1161/circresaha.114.301174 24855200 PMC4059102

[B35] Ridker P. M. Danielson E. Fonseca F. A. H. Genest J. Gotto A. M. Jr. Kastelein J. J. P. (2008). Rosuvastatin to Prevent Vascular Events in Men and Women with Elevated C-Reactive Protein. N. Engl. J. Med. 359, 2195–2207. 10.1056/NEJMoa0807646 18997196

[B36] Sisinni L. Pietrafesa M. Lepore S. Maddalena F. Condelli V. Esposito F. (2019). Endoplasmic Reticulum Stress and Unfolded Protein Response in Breast Cancer: The Balance between Apoptosis and Autophagy and its Role in Drug Resistance. Ijms 20, 857. 10.3390/ijms20040857 PMC6412864 30781465

[B37] Stein S. Schäfer N. Breitenstein A. Besler C. Winnik S. Lohmann C. (2010). SIRT1 Reduces Endothelial Activation without Affecting Vascular Function in ApoE-/- Mice. Aging 2, 353–360. 10.18632/aging.100162 20606253 PMC2919255

[B38] Steven S. Münzel T. Daiber A. (2015). Exploiting the Pleiotropic Antioxidant Effects of Established Drugs in Cardiovascular Disease. Ijms 16, 18185–18223. 10.3390/ijms160818185 26251902 PMC4581241

[B39] Suematsu N. Ojaimi C. Kinugawa S. Wang Z. Xu X. Koller Md A. (2007). Hyperhomocysteinemia Alters Cardiac Substrate Metabolism by Impairing Nitric Oxide Bioavailability through Oxidative Stress. Circulation 115, 255–262. 10.1161/circulationaha.106.652693 17200441

[B40] Suwaidi J. A. Hamasaki S. Higano S. T. Nishimura R. A. Holmes D. R. Jr. Lerman A. (2000). Long-term Follow-Up of Patients with Mild Coronary Artery Disease and Endothelial Dysfunction. Circulation 101, 948–954. 10.1161/01.cir.101.9.948 10704159

[B41] Tao J.-h. Zhao M. Wang D.-g. Yang C. Du L.-Y. Qiu W.-q. (2016). Biotransformation and Metabolic Profile of Catalpol with Human Intestinal Microflora by Ultra-performance Liquid Chromatography Coupled with Quadrupole Time-Of-Flight Mass Spectrometry. J. Chromatogr. B 1009-1010, 163–169. 10.1016/j.jchromb.2015.12.007 26741989

[B42] Ushio-Fukai M. Zafari A. M. Fukui T. Ishizaka N. Griendling K. K. (1996). p22 Is a Critical Component of the Superoxide-Generating NADH/NADPH Oxidase System and Regulates Angiotensin IIinduced Hypertrophy in Vascular Smooth Muscle Cells. J. Biol. Chem. 271, 23317–23321. 10.1074/jbc.271.38.23317 8798532

[B43] Varma-Doyle A. V. Lukiw W. J. Zhao Y. Lovera J. Devier D. (2021). A Hypothesis-Generating Scoping Review of miRs Identified in Both Multiple Sclerosis and Dementia, Their Protein Targets, and miR Signaling Pathways. J. Neurol. Sci. 420, 117202. 10.1016/j.jns.2020.117202 33183778

[B44] Xiang Z. Wang S. Li H. Dong P. Dong F. Li Z. (2021). Detection and Identification of Catalpol Metabolites in the Rat Plasma, Urine and Faeces Using Ultra-high Performance Liquid Chromatography-Q Exactive Hybrid Quadrupole-Orbitrap High-Resolution Accurate Mass Spectrometry. Cdm 22, 173–184. 10.2174/1389200221999201125205515 33243112

[B45] Xiong S. Patrushev N. Forouzandeh F. Hilenski L. Alexander R. W. (2015). PGC-1α Modulates Telomere Function and DNA Damage in Protecting against Aging-Related Chronic Diseases. Cel Rep. 12, 1391–1399. 10.1016/j.celrep.2015.07.047 PMC4549794 26299964

[B46] Xiong Y. Shi L. Wang L. Zhou Z. Wang C. Lin Y. (2017). Activation of Sirtuin 1 by Catalpol-Induced Down-Regulation of microRNA-132 Attenuates Endoplasmic Reticulum Stress in Colitis. Pharmacol. Res. 123, 73–82. 10.1016/j.phrs.2017.05.030 28655643

[B47] Yu X. Lv J. Zhu Y. Duan L. Ma L. (2013). Homocysteine Inhibits Hepatocyte Proliferation via Endoplasmic Reticulum Stress. PLoS One 8, e54265. 10.1371/journal.pone.0054265 23349842 PMC3551933

[B48] Zamzami N. Marzo I. Susin S. A. Brenner C. Larochette N. Marchetti P. (1998). The Thiol Crosslinking Agent Diamide Overcomes the Apoptosis-Inhibitory Effect of Bcl-2 by Enforcing Mitochondrial Permeability Transition. Oncogene 16, 1055–1063. 10.1038/sj.onc.1201864 9519879

[B49] Zhang J. Bi R. Meng Q. Wang C. Huo X. Liu Z. (2019). Catalpol Alleviates Adriamycin‐induced Nephropathy by Activating the SIRT1 Signalling Pathway *In Vivo* and *In Vitro* . Br. J. Pharmacol. 176, 4558–4573. 10.1111/bph.14822 31378931 PMC6932948

[B50] Zhang Y. Wang C. Jin Y. Yang Q. Meng Q. Liu Q. (2018). Activating the PGC-1α/TERT Pathway by Catalpol Ameliorates Atherosclerosis via Modulating ROS Production, DNA Damage, and Telomere Function: Implications on Mitochondria and Telomere Link. Oxidative Med. Cell Longevity 2018, 1–16. 10.1155/2018/2876350 PMC6036816 30046372

[B51] Zhu T. Zhang L. Ling S. Duan J. Qian F. Li Y. (2014). Scropolioside B Inhibits IL-1βand Cytokines Expression through NF-Κb and Inflammasome NLRP3 Pathways. Mediators Inflamm. 2014, 1–10. 10.1155/2014/819053 PMC4216717 25386048

